# Pharmacological agents for the prevention of colistin-induced nephrotoxicity

**DOI:** 10.1186/s40001-022-00689-w

**Published:** 2022-05-07

**Authors:** Mahtabalsadat Mirjalili, Ehsan Mirzaei, Afsaneh Vazin

**Affiliations:** 1 Department of Clinical Pharmacy, Faculty of Pharmacy, Shiraz University of Medical Sciences, Shiraz, Iran; 2 Department of Clinical Pharmacy, Faculty of Pharmacy and Pharmaceutical Sciences Research Center, Shahid Sadoughi University of Medical Sciences, Yazd, Iran

**Keywords:** Colistin, Nephrotoxicity, Nephroprotective, Antibiotics, Polymyxins

## Abstract

**Background:**

Colistin is a polymyxin antibiotic which has been used for treatment of Gram-negative infections, but it was withdrawn due to its nephrotoxicity. However, colistin has gained its popularity in recent years due to the reemergence of multidrug resistant Gram-negative infections and drug-induced toxicity is considered as the main obstacle for using this valuable antibiotic.

**Results:**

In total, 30 articles, including 29 animal studies and one clinical trial were included in this study. These compounds, including aged black garlic extract, albumin fragments, alpha lipoic acid, astaxanthin, baicalein, chrysin, cilastatin, colchicine, curcumin, cytochrome c, dexmedetomidine, gelofusine, grape seed proanthocyanidin extract, hesperidin, luteolin, lycopene, melatonin, methionine, N-acetylcysteine, silymarin, taurine, vitamin C, and vitamin E exhibited beneficial effects in most of the published works.

**Conclusions:**

In this review, the authors have attempted to review the available literature on the use of several compounds for prevention or attenuation of colistin-induced nephrotoxicity. Most of the studied compounds were potent antioxidants, and it seems that using antioxidants concomitantly can have a protective effect during the colistin exposure.

## Background

Polymyxins are a group of polypeptide antibiotics consisting of five drugs; i.e., polymyxin A–E. However, only polymyxin B and polymyxin E or colistin have therapeutic applications. Colistin was discovered in 1949. It is produced by a specific subspecies of *Bacillus polymyxa* called colistinus [1]. Colistin became available for clinical use in the 1969s. Approximately one decade later, however, it was replaced with less toxic antibiotics due to concerns for its adverse effects, particularly nephrotoxicity [2, 3]. Nonetheless, in recent years, the emergence of multidrug resistant Gram-negative bacilli (MDR-GNB), particularly *Pseudomonas aeruginosa*, *Acinetobacter baumannii*, and *Klebsiella pneumonia*, as well as the lack of development of new antibiotics have led to the increased use of colistin worldwide [4]. The most common side effects that limit the use of colistin are nephrotoxicity and neurotoxicity. Both side effects are dose-dependent and reversible, and permanent kidney damage has rarely been seen [5]. The incidence of colistin-induced nephrotoxicity has been found to range from 20 to 76% in different published studies [6]. Although the exact mechanism of colistin-induced nephrotoxicity is not known, it has been demonstrated that colistin use is associated with increased membrane permeability, oxidative injuries, and subsequently acute tubular necrosis [7]. Increased tubular epithelial cell membrane permeability results in cations, anions and water influx and subsequently cell swelling and lysis. Other mechanisms of nephrotoxicity include oxidative stress, apoptosis (via mitochondrial, death receptor, and endoplasmic reticulum pathways), cell cycle arrest, autophagy, altered nitric oxide balance, mitochondrial dysfunction and oxidative stress [6, 8, 9]. The factors affecting the increased risk of nephrotoxicity include age, sex, hypoalbuminemia, hyperbilirubinemia, concurrent nephrotoxic agents, various comorbidities, and high dose and long-term use of colistin [6]. Also, the administration of loading doses has increased the risk of colistin nephrotoxicity in some studies [10–12]. Colistin-induced nephrotoxicity has been associated with an increase in adverse outcomes in critically ill patients [13]. Considering the increasing incidence of MDR infections, a dearth of options of antimicrobial agents against MDR-GNB infection and the slow rate of antibiotic discovery, colistin remains an important option for severe MDR-GNB infections. However, its nephrotoxicity is a major clinical problem, affecting negatively its therapeutic use [14]. Thus, recognizing pharmacological interventions used to prevent or attenuate colistin-induced nephrotoxicity has gained special interest among clinicians, including physicians, pharmacists and other healthcare professionals in recent years.

The present study aims to review the available literature on the pharmacological interventions used to prevent or attenuate colistin-induced nephrotoxicity. These agents include antioxidant compounds, chemical agents, synthetic drugs, hormones, vitamins, and minerals.

## Methods

A literature review was conducted using the following electronic relevant databases: Scopus, PubMed, Medline, Embase, Cochrane Central Register of Controlled Trials, ISI Web of Knowledge, Cochrane Database of Systematic Reviews, and Google Scholar. The searched keywords included ‘Colistin, ‘Polymyxin E, ‘Nephrotoxicity’, ‘Kidney injury’, ‘Renal dysfunction’, ‘Renal impairment’, ‘Nephroprotective Agents’, ‘Nephroprotection’, ‘Nephroprotective effects’, ‘Nephroprotective activity’, and ‘Reno protective effects’. The references of published articles were also screened to find further relevant studies. Searches were performed by two authors independently to confirm consistency and accuracy of results. No time limitation regarding publication date was considered. Non-English language articles and congress abstracts were not eligible for the study.

In total, 28 articles have been included in this study. Both experimental and clinical studies evaluating the effects of the potential nephroprotective agents against colistin-induced nephrotoxicity are summarized in Table 1.

**Table 1:** Summary of the experimental and clinical studies regarding the effects of potential nephroprotective agents against colistin-induced nephrotoxicity

Agents	Subjects	Dose, route, and duration of administration	Dose, route, and duration of colistin administration	Parameters	Significant changes	References
Aged black garlic extract	Rats	1% of ABGE (100 µL per individual) injection intragastrically 30 min before colistin injection for 6 days	10 mg/kg of I.P. colistin for 6 consecutive days Injection was intragastrically done 30 min prior to colistin injection for 6 days	SCr, BUN, IL-1β TNFα, SOD, CAT, GSH, renal apoptosis (by TUNEL assay), ED1-positive cells infiltration, 8-OHdG, MDA, NF-κB, INOS, COX-2, TGF-β1, renal histology	Prevented colistin-induced elevation of BUN and SCr, reduced TUNEL- and CD68, suppressed oxidative stress and inflammatory biomarkers including 8-OHdG, MDA, NF-κB, iNOS, COX-2, TGF-β1, IL-1β, and TNF-α., restored antioxidant levels such as renal SOD, CAT, and GSH, and alleviated tubular damage including vacuolation and necrosis	[20]
Albumin fragments	Rats	50 mg/kg I.P. before the injection of colistin	1.0 mg/kg of I.V. colistin sulfate	Urine NAG, urinary colistin excretion	Decreased urinary NAG excretion and increased the urinary excretion of colistin	[26]
Alpha-lipoic acid	Rats	100 mg/kg I.P. 30 min before the administration of CMS for 10 days	450.000 IU/kg/day of I.P. CMS for 10 days	Renal MDA, SOD, TNF-α, urine KIM-1, urine microalb/Cr, Scr, MRNA expression of KIM-1, Nox4, and p22phox in the kidney, kidney active caspase-3 protein expression	Attenuated renal injury, decreased urine KIM-1, mRNA expression of KIM-1, Nox4d and p22phox in the kidney, and kidney active caspase-3 protein expression	[35]
Astaxanthin	Rats	20 mg/kg/day given by oral gavages for seven days	300,000 or 450,000 IU/kg/day of I.M. CMS in twice daily doses for seven days	Plasma/urine Cr, Urine GGT, MDA, SOD, CAT, GPx, GSH, and renal histology	Attenuated PCr and urine GGT levels, partially diminished the degree of renal tissue damage induced by colistin	[43]
Baicalein	Mice	25, 50, and 100 mg/kg/day orally for seven days	18 mg/kg/day of I.P. colistin (sulphate) for 7 days	Serum BUN, SCr, MDA, NO, GSH, SOD, CAT, iNOS, Caspase-3, Caspase-9, TNF-a, IL-1β, MRNA expression of Nrf2, HO-1, and NF-kB, histopathological changes	Attenuated colistin-induced tissue damage, decreased BUN, SCr, IL-1b, and TNF-a levels, attenuated all the colistin-induced biomarkers of oxidative stress including MDA, iNOS, NO, SOD, and CAT, upregulated the expression of Nrf2 and HO-1 mRNAs, and downregulated the expression of NF-κB mRNA	[49]
Chrysin	Rats	50 mg/kg/day orally for 7 days	73 mg/kg I.M. colistin: 1.0 and 2.0 mg/kg on day 1; 2.50 mg/kg twice daily on day 2; 3.5 and 5.5 mg/kg on day 3; 8.0 mg/kg twice daily on days 4, 5, and 6; and 8.0 mg/kg on day 7	Serum urea, SCr, MDA, GSH, SOD, CAT, GPx, TNF-α, IL-6, IL-1β, Cystatin C, and calbindin D28K immunopositivities, injuries to the proximal and distal tubules	Decreased MDA, TNF-α, IL-6, and IL-1β, increased GSH, SOD, CAT, GPx, cystatin C, and calbindin D28K immunopositivities, alleviated renal injury	[56]
Cilastatin	Mice	100 mg/kg /day for 4 days	30 mg/kg/day of S.Q. colistin for 4 days	Urinary NAG, KIM-1 expression in kidney tissue, tubular injury (morphologic changes)	Decreased urinary NAG and KIM-1 expression in proximal tubule epithelial cells and suppressed colistin-induced tubulointerstitial injury	[23]
Colchicine	Rats	3.5 mg/kg I.P. before the injection of colistin	1.0 mg/kg of I.V. colistin sulfate	Urine NAG	Decreased urinary NAG excretion	[26]
Curcumin	Rats	200 mg /kg/day orally for 6 days	300,000 IU/kg/day of I.P. CMS IP for 6 days	Serum urea, SCr, Serum UA, GSH, MDA, CAT, NO, TNF-α, IL-6, Bcl-2, Caspase-3, histopathological changes	Partially restored altered biochemical markers including increased SCr, serum urea, and UA, MDA, NO, TNF-α, IL-6, and caspase-3 expression levels and decreased CAT, GSH, and Bcl-2 expressions and alleviated histopathological changes	[65]
Cytochrome c	Rats	100 mg/kg I.P. before the injection of colistin	1.0 mg/kg of I.V. colistin sulfate	Urine NAG, urinary colistin excretion, inhibitory effect on the binding of colistin to megalin	Decreased urinary NAG excretion, increased the urinary excretion of colistin, inhibited the binding of colistin to megalin competitively	[26]
Dexmedetomidine	Rats	10 and 20 mcg/kg I.P. twice a day 20 min before the injection of colistin for seven days	10 mg/kg of I.P. CMS	BUN, SCr, KIM-1, TAS, TOS, caspase-3	Decreased BUN, Cr, and TOS	[71]
Gelofusin	Mice	Cumulative I.P. doses of 450, 900, 1,800, and 3,600 mg/kg: 75, 150, 300, and 600 mg/kg every 2 h (6 doses)	S.Q. colistin sulfate at a cumulative dose of 84 mg/kg: 14 mg/kg every 2 h (6 doses)	Renal histology	Ameliorated colistin-induced nephrotoxicity in a dose-dependent manner	[75]
Grape seed proanthocyanidin extract	Rats	100 mg/kg/day orally	300,000 IU/kg/day of I.P. CMS for 7 days	BUN, SCr, TOS, TAS, MDA, OSI, Caspase 1, Caspase 3, Calpain 1, iNOS, eNOS, renal apoptosis (TUNEL assay), histopathological changes	Decreased BUN, SCr, renal histopathological scores, TUNEL, caspase 1 and 3, calpain 1, iNOS, and eNOS staining	[79]
Hesperidin	Rats	300 mg/kg/day orally for 7 days	73 mg/kg I.M. colistin: 1.0 and 2.0 mg/kg on day 1; 2.50 mg/kg twice daily on day 2; 3.5 and 5.5 mg/kg on day 3; 8.0 mg/kg twice daily on days 4, 5, and 6; and 8.0 mg/kg on day 7	MDA, GSH, SOD, CAT, GPx, serum urea, SCr, TNF-α, IL-6, IL-1β, Cystatin C, and calbindin D28K immunopositivities and injuries to the proximal and distal tubules	Decreased MDA, TNF-α, IL-6, and IL-1β, increased GSH, SOD, CAT, GPx, cystatin C, and calbindin D28K immunopositivities, and alleviated renal injury	[56]
Luteolin	Rats	10 mg/kg I.P 4 h before colistin administration for 7 days	480,000 IU/kg/day of IP colistin for 7 days	Renal histology	Decreased the number of apoptotic cells and renal histological damage score	[94]
Lycopene	Mice	5 and 20 mg /kg/day orally 2 h before colistin administration for 7 days	15 mg/kg/day of I.V. colistin sulfate in two doses via a 3-min infusion for 7 days	BUN, SCr, MDA, NO, iNOS, GSH, SOD, CAT, MDA, caspase-3, caspase-9, HO-1, MRNA expression of OH-1, Nrf2, and NF-κB in the kidney, and histopathological changes	Increased levels of GSH, SOD, and Cat, decreased concentrations of BUN and SCr, caspase-dependent tubular apoptosis/necrosis, MDA, NO, iNOS, and HO-1 activity, downregulated the mRNA expression of NF-κB, and upregulated the mRNA expression of Nrf2 and HO-1 mRNA	[97]
Melatonin	Rats	5 mg/kg I.V. twice a day 20 min prior to each colistin dose for 7 days	36.5 mg/kg of I.V. colistin sulfate: 0.5 and 1.0 mg/kg on day 1; 1.25 mg/kg twice daily on day 2; 1.75 and 2.75 mg/kg on day 3; 4.0 mg/kg twice daily on days 4, 5, and 6; and 4.0 mg/kg on day 7	Urine NAG, PCr, SOD, renal histology	Mitigated the consequences of colistin-induced renal injury including increased urine NAG and PCr as well as renal histological abnormalities	[103]
N-Acetyl cysteine	Rats	150 mg/kg/day I.P. for 6 days	300,000 IU/kg/day of I.P. colistin (CMS)	BUN, PCr, UCr, ClCr, urine protein, plasma TNF-α, SOD, MDA, e-NOS, i-NOS, NT-3	Reduced renal tissue SOD level and reversed immunocytochemical staining of i-NOS and NT-3	
N-Acetyl cysteine	Rats	300 mg/kg/day I.P in two divided doses for 10 days	300,000 IU/kg/day of I.P. CMS for 10 days	SCr, urine NAG, TOS, TAS, OSI, eNOS, SOD2, MMP, renal apoptosis (by TUNEL assay), renal histology examination	Reversed colistin-induced negative effects, as determined by increased SCr, urine NAG, apoptosis index, and renal histological damage score as well as by decreased renal expression levels of eNOS, SOD2, and MMP	[110]
Silymarin	Rats	50 mg/kg I.V silymarin twice a day 2 h before polymyxin E injection for 7 days	A cumulative dose of 36.5 mg/kg I.V. polymyxin E given twice a day, 8 h apart, for 7 days	Histological, ultrastructural, and morphometric changes	Alleviated the degenerative changes on the rat kidney induced by polymyxin E	[111]
Silymarin	Rats	50 mg/kg I.V silymarin twice a day 2 h before polymyxin E injection for 7 days	A cumulative dose of 36.5 mg/kg I.V. polymyxin E given twice a day, 8 h apart, for 7 days	SCr, serum urea, serum UA, serum Na, serum K,	ameliorated the biochemical changes induced by polymyxin E in rats including elevated urinary NAG and serum levels of urea, creatinine, uric acid, sodium, and potassium. However, the differences between polymyxin and polymyxin + silybin groups were statistically significant only for NAG	[117]
Silymarin	Rats	100 mg/kg/day in two divided doses given by oral gavages for 7 days	750.000 IU/kg/day of I.P. colistin in two divided doses for seven days	SCr, Cystatin C, GPx, SOD, MDA, Renal apoptosis (by TUNEL assay), histopathological changes	Increased GPx and SOD and made some improvements in tubular necrosis	[118]
Taurine	Mice	500 and 1000 mg/kg, IP)	15 mg/kg/day of I.V. colistin for 7 days	PCr, BUN, renal and mitochondrial GSH, renal and mitochondrial GSSG, renal and mitochondrial LPO, renal FRAP, mitochondrial dehydrogenases activity, mitochondrial depolarization, mitochondrial ATP, mitochondrial swelling and permeabilization, histopathological changes	Decreased colistin-induced elevation in plasma Cr and BUN, reversed colistin-induced negative effects such as increased renal ROS, LPO, and GSSG, mitochondrial LPO, permeabilization, and GSSG content, and decreased renal TAC, GSH stores, mitochondrial dehydrogenase activity, membrane potential, GSH, and ATP	[119]
Vitamin C	Rats, rat proximal tubular cells (NRK-52E)	50 or 200 mg/kg I.V. twice daily 20 min before each colistin dose for 7 day	36.5 mg/kg of I.V. colistin sulfate: 0.5 and 1.0 mg/kg on day 1; 1.25 mg/kg twice daily on day 2; 1.75 and 2.75 mg/kg on day 3; 4.0 mg/kg twice daily on days 4, 5, and 6; and 4.0 mg/kg on day 7	PCr, urine NAG, SOD, renal apoptosis (by TUNEL assay), renal histology	Decreased SCr, urine NAG, and histological abnormalities and had a dose-dependent inhibitory effect on colistin-induced apoptosis	[123]
Vitamin C	Humans	2 g I.V. every 12 h 20 min before CMS administration	CMS at a loading dose of 300 mg of CBA followed by renally adjusted maintenance doses every 12 h	SCr, CrCl, AKI, urine NGAL, urine NAG, clinical outcome, microbiological outcome, mortality, plasma colistin concentrations	No significant differences	[128]
Vitamin E	Rats	100 mg/kg/day given by oral gavage for 7 days	300,000 and 450,000 IU/kg/day of I.M. CMS in twice daily doses for 7 days	PCr, UCr, urine GGT, MDA, SOD, CAT, GPx, GSH, renal histology	Attenuated PCr and urine GGT levels and partially diminished the degree of colistin-induced renal damage	[129]
Vitamin E	Rats	100 mg/kg/day for 2 weeks after colistin discontinuation	300,000 and 450,000 IU/kg/day of I.M. CMS in twice daily doses for 7 days	PCr, UCr, urine NAG, MDA, SOD, GSH, renal histology	Attenuated increased NAG and MDA levels, attenuated decreased SOD and GSH activities, and improved tubular regeneration	[43]
Vitamin E + Vitamin C	Rats	100 mg/kg/day given by oral gavages for 7 days	450,000 IU/kg/day of CMS for 7 days	Urine NAG, urine GGT, PCr, plasma level of vitamins E and C, MDA, SOD, CAT, GPx, renal histology	Restored all biochemical parameters (increased NAG GGT and MDA and decreased the plasma levels of vitamin E and C, SOD, CAT, and GPx, and improved histopathological damage	[133]
Vitamin E	Humans	400 mg vitamin E in form of alpha tocopherol daily	–	–	–	[134]

### Aged black garlic extract

Aged black garlic extract (ABGE) is produced by storing fresh garlic in an aqueous ethanol solution for up to 20 months and contains antioxidant compounds that prevent oxidative damage. The process of prolonged extraction modifies unstable compounds such as allicin and converts them to more stable and bioavailable water-soluble organosulfur phytochemicals such as S-allylcysteine and S-allylmercaptocysteine [15]. ABGE has the highest pharmacological and antioxidant activities in comparison to other garlic preparations [16]. Currently, therapeutic and health-promoting effects of ABGE such as antioxidant activities, reactive oxygen species (ROS) scavenging properties, lipid peroxidation inhibition, endothelial cells protection, inhibition of the activation of nuclear factor kappa-light-chain-enhancer of activated B cells (NF-κB), antihypertensive, anti-thrombotic, neuroprotective, antiaging, cholesterol lowering, and anti-diabetes properties, prevention of deoxyribonucleic acid (DNA) damage and mutagenesis, anti-cancer activity, and immunity modulation have attracted considerable interest [17, 18]. The beneficial effects of ABGE on the amelioration of the toxic effects of cisplatin, doxorubicin, acrylamide, carbon tetrachloride, acetaminophen, and cadmium have been previously proven [17, 19]. Shin et al*.* investigated the reno protective effect of ABGE against colistin toxicity in rats, which was attributed to its antioxidant and anti-inflammatory properties. The results indicated that 1% ABGE (100 µL per individual) injection intragastrically 30 min prior to 10 mg/kg colistin administration for six consecutive days prevented the elevation of the serum levels of blood urea nitrogen (BUN) and creatinine (Cr). Pretreatment with ABGE could also alleviate tubular damage including vacuolation and necrosis, reduce terminal deoxynucleotidyl transferase dUTP nick end labeling (TUNEL) and CD68 positive cells, suppress oxidative stress and inflammatory biomarkers including 8-hydroxydeoxyguanosine (8-OHdG), malondialdehyde (MDA), NF-κB, inducible nitric oxide synthase (iNOS), cyclooxygenase-2 (COX-2), transforming growth factor-beta 1 (TGF-β1), interleukin (IL)-1β, and tumor necrosis factor-alpha (TNF-α), and restore antioxidant levels such as renal superoxide dismutase (SOD), catalase (CAT), and reduced glutathione [20].

### Albumin fragments

The membrane-associated endocytic receptor megalin is an extremely large glycoprotein (≈ 600 kda), belonging to the low-density lipoprotein receptor superfamily. Megalin is heavily expressed on the apical membrane of proximal tubule epithelial cells (PTECs) and plays an important role in the receptor-mediated endocytosis and the subsequent metabolization of proteins and nutrients [21]. Megalin mediates the proximal tubular uptake of different groups of ligands including plasma proteins, peptides, enzymes, vitamin-binding proteins, hormones, hormone-binding proteins, drugs, and toxins. Albumin, myoglobin, hemoglobin, vitamin D-binding protein, retinol-binding protein, β2-microglobulin, lactoferrin, and insulin have been identified as megalin ligands. Additionally, some nephrotoxic drugs such as aminoglycosides, cisplatin, vancomycin, polymyxin B, and colistin are absorbed through megalin receptors [22, 23].

Albumin, a protein with a molecular weight of 67 kDa, is a ligand for megalin and its reabsorption in renal proximal tubules is mediated through megalin. Thus, it has been assumed that it can be used for the inhibition of the renal accumulation of colistin [24]. Considering the fact that only a small fraction of albumin passes the glomerular membrane, albumin fragments can be a more appropriate alternative for the inhibition of colistin accumulation in the kidneys [25]. The results of the research by Suzuki et al*.* indicated that the co-administration of albumin fragments (with molecular masses of less than 50 kDa) at the dose of 50 mg with 1.0 mg/kg colistin resulted in a significant decrease in urinary N-acetyl-β-D glucosaminidase (NAG) excretion as well as an increase in urinary colistin excretion [26].

### Alpha-lipoic acid

Alpha-lipoic acid (ALA), also known as thioctic acid, is an organosulfur compound commonly found in mitochondria, which is necessary for different enzymatic functions. Accumulating evidence has suggested that ALA has multiple pharmacological effects including antidiabetic, anti-dementia or anti-Alzheimer’s disease (AD), anti-ageing, metal chelating and detoxifying, anti-inflammatory, anti-cancer, cardiovascular protective, cognitive protective, and neuroprotective properties [27]. It has also been discovered in various clinical trials that ALA can be efficient in particular diseases including diabetic neuropathy, obesity, schizophrenia, multiple sclerosis (MS), pregnancy complications, and organ transplantation [27]. The nephroprotective actions of ALA against nephrotoxicity induced by cisplatin, chloroquine, methotrexate, and iron overload have been shown in different studies, as well [28–33]. Moreover, the great antioxidant potential and scavenging activity of ALA can protect against oxidative injuries [34].

Oktan et al*.* investigated ALA’s ability to protect against colistin-induced nephrotoxicity in rats. In that study, the rats received 100 mg/kg ALA 30 min before the administration of 450,000 IU/kg colistimethate sodium (CMS) for 10 days. The results demonstrated that ALA could reverse the effects of colistin-induced nephrotoxicity by reducing oxidative stress and renal tubular apoptosis, partly through its suppressing effect on NADPH oxidase 4 (Nox4) and caspase-3. In addition, messenger ribonucleic acid (mRNA) expression of kidney injury molecule-1 (KIM-1), Nox4, and p22phox in the kidney as well as kidney active caspase-3 protein expression were reduced by the administration of ALA. ALA administration also resulted in a significant reduction in the level of urine KIM-1, as a strong biomarker for tubular injury. However, there were no significant changes in renal MDA, SOD, TNF-α, urine microalbumin/Cr, and serum Cr [35].

### Astaxanthin

Astaxanthin, a naturally occurring lipid-soluble and red–orange oxycarotenoid pigment, is found in several species of bacteria and yeasts and a wide variety of aquatic organisms such as microalgae, fish, and crustaceans such as shrimps [36]. Its potential pharmacological effects including antioxidant properties, DNA repair, cell regeneration, and neuroprotective, immunomodulatory, antiproliferative, anti-inflammatory, anti-apoptotic, antidiabetic, anticancer, photoprotective, and skin-protective effects have been established in various investigations [37–39]. Furthermore, it can prevent oxidative damage to fatty acids and biological membranes by scavenging lipid radicals and destroying peroxides [40]. Animal studies have also revealed its nephroprotective effects against diabetic nephropathy and mercuric chloride-induced nephrotoxicity [41, 42].

An experimental study conducted by Ghlissia et al*.* exhibited that the co-treatment of astaxanthin (20 mg/kg/day given by oral gavages) and colistin (300,000 or 450,000 IU/kg/day of intramuscular (IM) CMS) for 7 days provided nephroprotection against colistin evidenced by biochemical, histological, and oxidative stress parameters. Besides, astaxanthin prevented change in antioxidant parameters including SOD, glutathione peroxidase (GPx), CAT, reduced glutathione (GSH), and MDA, induced by colistin. It also attenuated the plasma levels of Cr and urine Gamma-Glutamyl Transferase (GGT) and partially diminished the degree of renal tissue damage induced by colistin [43].

### Baicalein

Baicalein is a flavone and an active component in the root of Scutellaria baicalensis [44]. There is a large body of evidence on its beneficial pharmacological activities including anti-inflammatory, antioxidative, antiviral, antibacterial, immuno-regulatory, anti-cancer, cardioprotective, neuroprotective, and hepatoprotective effects [45–47]. Due to its anti-inflammatory, anti-apoptotic, and antioxidant effects, baicalein was used as a nephroprotective agent against cisplatin and myocardial ischemia-induced nephrotoxicity in the previous studies [47, 48].

In an experimental study, Dai et al*.* assessed the effects of baicalein on the colistin-induced changes in the renal tissue of a mouse model exposed to 18 mg/kg/day colistin. The results indicated that the 7-day treatment with oral baicalein at 25, 50, and 100 mg/kg/day doses could attenuate colistin-induced tissue damages, decrease BUN, SCr, IL-1β, and TNF-a levels, attenuate all the colistin-induced biomarkers of oxidative stress including MDA, iNOS, nitric oxide (NO), SOD, and CAT, upregulate the expression of nuclear factor-erythroid 2-related factor 2 (Nrf2) and heme oxygenase-1 (HO-1) mRNAs, and downregulate the expression of NF-κB mRNA in a dose-dependent manner [49].

### Chrysin

Chrysin, as a natural flavonoid, is considered one of the main metabolites of medicinal plants and is found in many fruits, vegetables, and plant extracts such as honey, blue passion flower, Passiflora caerulea, and propolis [50]. The antioxidant, anti-inflammatory, anticancer, antivirus, anti-diabetogenic, anti-anxiolytic, and anti-autophagic effects of chrysin have been proved in various studies [51–53]. Its beneficial effects have also been reported in paracetamol-induced nephrotoxicity and doxorubicin-induced cardiotoxicity [54, 55]. Moreover, the beneficial effect of chrysin on the prevention of colistin-induced renal injury was shown in an animal study. In that study, a cumulative dose of 73 mg/kg of colistin was administered to rats during 7 days. Then, oral chrysin was administered at 25 and 50 mg/kg doses for 7 days. The results demonstrated that chrysin could alleviate colistin-induced renal inflammation by significantly decreasing the levels of TNF-α, IL-6, IL-1β, and MDA as well as increasing the levels of SOD, CAT, GPx, GSH, cystatin C (Cys C), and calbindin D28K. Additionally, histopathological examination revealed an improvement in colistin-induced interstitial nephritis in chrysin-treated rats [56].

### Cilastatin

Cisplatin, an inhibitor of renal dehydropeptidase-I (DHP-I), has been co-administered with imipenem to prevent its metabolism through DHP-I of proximal renal tubules, increase its stability, and suppress the tubular injury. Its nephroprotective effects have also been proved against nephrotoxicity induced by other drugs such as vancomycin, cisplatin, cyclosporine, tacrolimus, diclofenac, and gentamycin [57]. Considering the fact that megalin and DHP-1 are both located at the brush border membranes of [58], cilastatin may encounter megalin in vivo [23].

Hori et al*.* emphasized the protective role of colchicine against colistin-induced nephrotoxicity, which was achieved through megalin blockade. Importantly, subcutaneous (SQ) injection of 100 mg/kg cilastatin with 30 mg/kg colistin once daily for 4 days decreased the urinary excretion of NAG and the expression of (KIM-1 in proximal tubule epithelial cells and suppressed colistin-induced tubulointerstitial injury including tubular vacuolization, tubular dilation or atrophy, brush border loss, tubular cell lysis, and cast formation [23].

### Colchicine

Previous studies have demonstrated that microtubules are involved in the apical accumulation of megalin-containing vacuoles [59]. Colchicine, a microtubule-depolymerizing agent, interferes with intracellular transport of megalin from the cell membrane and causes the internalization of megalin from the brush border membrane into intracellular vesicles [60]. In a study carried out on colistin-treated rats, the effect of colchicine, a microtubule-depolymerizing agent, was assessed on colistin-induced nephrotoxicity. The results demonstrated that colchicine administration (3.5 mg/kg, IP) before the injection of 1.0 mg/kg IV colistin led to a significant decrease in urinary NAG excretion [26].

### Curcumin

Curcumin, a polyphenol compound, is found within turmeric and is extracted from the rhizome of Curcuma longa Linn. Curcumin has broad biological activities including anti-inflammatory, antioxidant, and anticancer effects, inhibition of angiogenesis and metastasis, radio sensitization in cancer cells, radioprotection in normal cells, and anti-human immunodeficiency virus, antibacterial, nematocidal, and hepatoprotective functions [61]. Its renoprotective effects have also been proved in several experiments including diabetic nephropathy, chronic renal failure, renal injury induced by ischemia and reperfusion, shock-wave lithotripsy, and nephrotoxicity induced by compounds such as cisplatin, methotrexate, gentamicin, adriamycin, chloroquine, iron nitrilotriacetate, sodium fluoride, heavy metals, and triiodothyronine [62–64]. It seems that curcumin’s pleiotropic activities are related to its free radical scavenging, hydrogen donation, and antioxidant properties as well as to the activation of signaling pathways such as NF-kB, protein kinase B (Akt), and Nrf2 [62].

Edrees et al*.* evaluated the nephroprotective effects of the oral administration of curcumin (200 mg/kg/day for 6 days) on antioxidant, inflammatory, and apoptotic markers as well as on colistin-induced renal damage in rats treated with 300,000 IU colistin/kg/day. They found that curcumin treatment could partially restore the altered biochemical markers such as increased expression levels of SCr, serum urea, serum uric acid (UA), MDA, NO, TNF-α, IL-6, and caspase-3 as well as decreased expression levels of CAT, GSH, and B-cell lymphoma 2 (Bcl-2). Furthermore, co-treatment with curcumin could alleviate the renal histopathological findings. Curcumin was found to exert its protective effects on the renal tissue via its antioxidant, anti-inflammatory, and anti-apoptotic activities [65].

### Cytochrome C

Cytochrome C (cyt c) is a small mitochondrial protein (MW = 12 kDa), which is involved in the mitochondrial respiratory chain and acts downstream of p53 in the apoptotic pathway. It has been used for the treatment of carbon monoxide poisoning, hypnotic intoxication, severe hypoxia in shock, dyspnea, myocardial anoxia, and cancers [66].

A study conducted by Suzuki et al*.* evaluated the effect of cyt c, as a megalin ligand, on colistin-induced nephrotoxicity as well as on the binding of colistin to megalin. Co-administration of 1.0 mg/kg colistin with 100 mg/kg cyt c significantly decreased the urinary NAG excretion, a marker of renal tubular damage, and increased the urinary excretion of colistin. Furthermore, cyt c inhibited the binding of colistin to megalin competitively [26].

### Dexmedetomidine

Dexmedetomidine is a potent and highly selective α2-adrenoceptor agonist with sedative, analgesic, sympatholytic, hemodynamic stabilizing, anti-inflammatory, and diuretic activities [67]. In recent years, numerous studies have been focused on the organoprotective properties of dexmedetomidine in brain, heart, and kidneys. The α2 adrenoceptors are distributed widely in proximal and distal tubules, as well as in peritubular vasculature. The underlying mechanism of its nephroprotection may be attributed to its anti-inflammatory, anti-oxidative stress, and anti-apoptotic actions. Moreover, it causes renal artery vasodilatation and an increase in renal blood flow and urine output through the activation of adrenoreceptors, inhibition of renal sympathetic nerves, reduction of the norepinephrine level and vasopressin, increase of atrial natriuretic peptide secretion, and inhibition of renin release [68]. The renoprotective effects of dexmedetomidine have been supported in animal and human models of renal injury induced by ischemia–reperfusion or sepsis and following cardiac surgery [69, 70].

In a study by Talih et al*.* the effect of dexmedetomidine on colistin nephrotoxicity was assessed in rats by determining the plasma levels of BUN, Cr, KIM-1, total oxidative stress (TOS), and total anti-oxidative stress (TAS). The altered parameters were improved using 10 and 20 mcg/kg IP dexmedetomidine 20 min before the administration of 10 mg/kg IP colistin twice a day for 7 days. Moreover, the apoptotic activity was evaluated via caspase-3 immunostaining, which indicated that the caspase-3 staining rate was lower in the dexmedetomidine group compared to the colistin group, but the difference was not statistically significant [71].

### Gelofusine

Gelofusine is a 4% w/v solution of succinylated bovine gelatin (modified fluid gelatin) with a mean molecular mass of 30,000 Da, which is used for volume replacement. It has been demonstrated that gelofusine can competitively inhibit the renal tubular reabsorption of proteins and peptides such as albumin, β2-microglobulin, nephrotoxic radiolabeled integrin, and somatostatin peptides [72–74]. The exact mechanism for this inhibition is not completely understood, but it is proposed to be related to the megalin receptor. Considering the role of megalin in the renal reabsorption of colistin, it has been proposed that gelofusine can competitively inhibit its renal reabsorption and endosomal/lysosomal trafficking [75].

Sivanesan et al*.* evaluated the nephroprotective effects of gelofusine in a mouse model of colistin-induced nephrotoxicity using a fixed dose of colistin (cumulative dose, 84 mg/kg SC) and four different gelofusine dosage regimens (cumulative doses of 450, 900, 1800, and 3600 mg/kg IP). The results indicated that gelofusine could serve as a safe adjunct for ameliorating nephrotoxicity and increasing the therapeutic index of polymyxins at the histological level in a dose-dependent manner. The dose-dependent nephroprotective effect of gelofusine could be associated with competition between gelofusine and colistin for uptake by proximal tubular cells [75].

### Grape seed proanthocyanidin extract

Grape seed proanthocyanidin extract (GSPE) is a natural flavonoid polyphenolic molecule obtained from black grape seeds. GSPE is one of the most potent plant antioxidants and free radical scavengers whose pharmacological benefits including antioxidant, antiapoptotic, antimicrobial, anticarcinogenic, vasodilator, anti-fatigue, and anti-inflammatory properties have been demonstrated in numerous investigations [76]. GSPE has been found to be effective in a broad spectrum of pathological conditions including cardiovascular diseases, acute and chronic stress, gastrointestinal distress, neurological disorders, pancreatitis, neoplastic processes, and carcinogenesis, drug and chemical-induced multi-organ toxicity, metabolic syndrome-related disorders, and obesity. Its nephroprotective effects have also been proved in renal injury induced by cadmium, contrast agents, carboplatin, thalidomide, amikacin, cyclosporine A, and diabetes [77–79].

Ozkan et al*.* reported the protective role of GSPE (100 mg/kg/day orally along with 300,000 IU/kg/day IP colistimethate sodium for 7 days) against colistin-induced nephrotoxicity, including a significant decrease in BUN and Cr levels, renal histopathological scores, TUNEL, caspase 1 and 3, calpain 1, iNOS, and endothelial NO synthase [80] staining. The results suggested that GSPE exerted its renoprotective effects via reducing oxidative damage, caspase mediated apoptosis, and iNOS, which might be involved in the pathogenesis of colistin-associated nephropathy [79].

### Hesperidin

Hesperidin is a flavonoid glycoside, which is present in high concentrations in some citrus species such as lemon, sweet orange, and grapefruit [81]. Previous studies indicated that hesperidin had antioxidant, anti-inflammatory, antihypertensive, lipid-lowering, insulin sensitivity, bone loss inhibitory, and neuroprotective effects [81–84]. Hesperidin has also shown promising results in acetaminophen toxicity, cisplatin-induced acute kidney injury, and lipopolysaccharide-caused lung injury in animal studies [85–87]. Hesperidin’s pharmacologic effects have been attributed to its significant free radical scavenging and anti-oxidation activities [88].

In an animal study, treatment with hesperidin was associated with protective effects on renal injury induced by colistin in rats. Based on the results, oral hesperidin administration at 200 and 300 mg/kg/day doses for 7 days after colistin administration at a cumulative dose of 73 mg/kg could reduce TNF-α, IL-6 IL-1β, and MDA levels. It also increased the activities of SOD, CAT, and GPx as well as the level of GSH. Furthermore, Cys C and calbindin D28K immunopositivities were significantly improved through hesperidin treatment [56].

### Luteolin

Luteolin, a 3,4,5,7-tetrahydroxyflavone, is the main member of the flavonoid family that is found in numerous plants, fruits, and vegetables. Luteolin has major biological and pharmacological properties including antioxidant, anti-inflammatory, antiapoptosis, neuroprotective, and cardioprotective activities [89]. Its notable defensive and preventive effects against pathological conditions such as chronic inflammatory diseases, atherosclerosis, drug-induced liver injury, diabetes, cancer, and allergies have also attracted attention in clinical practice [90]. Regarding its nephroprotective effects, luteolin has been found to protect against cisplatin, bisphenol-A, lead, chromium, mercury-induced nephrotoxicity, diabetic nephropathy, lipopolysaccharide (LPS) induced acute renal injury, and so forth [91–93].

In an animal experiment, Arslan et al*.* studied the potential effects of luteolin (10 mg/kg) on the biomarkers of nephrotoxicity in rats receiving 480,000 IU/kg/day colistin IP for 7 days. The findings showed that the level of post-treatment Cr was significantly increased in the rats receiving colistin alone, while no significant difference was observed in pretreatment and post-treatment Cr levels in the group receiving colistin + luteolin. Besides, the number of apoptotic cells and renal histological damage score were significantly higher in the colistin group compared to the rats receiving colistin in combination with luteolin [94].

### Lycopene

Lycopene is a lipophilic pigment, which belongs to the carotenoid family and is responsible for the red color of tomato and its related products. It is considered as an important natural antioxidant, anti-inflammatory agent, and free radical scavenger, which protects cell biomolecules including lipids, lipoproteins, and DNA against oxidation [95]. Up to now, numerous studies have evaluated the effects of lycopene in chronic diseases such as certain types of cancer (e.g., lung and prostate), male infertility, AD, chronic obstructive pulmonary diseases (COPD), osteoporosis, and neuropathic pain [96]. The beneficial roles of lycopene in the prevention of nephrotoxicity induced by gentamicin, vancomycin, adriamycin, cisplatin, mercuric chloride, combined use of isoniazid and rifampicin, furan, and ischemic reperfusion have been reported [97–99].

Dai et al*.* studied the effect of lycopene on colistin-induced nephrotoxicity in a mouse model. Lycopene pretreatment (5 or 20 mg/kg/day orally) 2 h before the administration of colistin (15 mg/kg/day intravenously) for 7 days attenuated colistin-induced nephrotoxicity through increasing the levels of endogenous antioxidant biomarkers including GSH, SOD, and CAT as well as decreasing the concentrations of BUN and SCr, caspase-dependent tubular apoptosis/necrosis, MDA, NO, iNOS, and HO-1 activity. Furthermore, lycopene, especially in the colistin plus lycopene (20 mg/kg) group, was found to significantly downregulate the expression of NF-κB mRNA and upregulate the expressions of Nrf2 and HO-1 mRNA, which seem to be the main reason for its preventive effects on colistin-induced nephrotoxicity. This study was the first to reveal that the Nrf2/HO-1 pathway plays a protective role in colistin-induced nephrotoxicity in mice [97].

### Melatonin

Melatonin, N-acetyl-5-methoxytryptamine, is mainly synthesized within the pineal cells and is released into the circulation in a circadian rhythm. It plays important roles in complex physiological and pathological processes through its receptors, which are located in most central and peripheral tissues and organs including kidneys. Melatonin and its metabolites have antioxidant and direct free radical scavenging activities, resulting in the augmentation of antioxidant enzymes in oxidative stress. Its beneficial effects also result from its ability to inhibit principle pro-inflammatory or apoptotic cytokines such as TNF-α and NF-κB [100]. Melatonin was first used for the treatment of sleep disorders and jet lags. Recently, however, researchers have found new applications for this hormone including cancer, neurodegenerative and mental disorders (e.g., AD), immune disorders (including rheumatoid arthritis), endocrine and metabolic disorders (including type II diabetes), mental and neurodegenerative disorders, and cardiovascular diseases [101]. The nephroprotective capacity of melatonin has also been elucidated in different studies, revealing it as a promising agent for the pathogenetic correction of diabetic nephropathy, chronic kidney disease progression, idiopathic membranous nephropathy, ischemia–reperfusion, and rhabdomyolysis-induced renal injury as well as the eradication of renal oxidative stress induced by different agents such as anticancers (cisplatin, ifosfamide, doxorubicin, methotrexate, and mechlorethamine), antibiotics (gentamicin, amikacin, ciprofloxacin, vancomycin, and tenofovir), immunosuppressants (cyclosporine and tacrolimus), antipyretics (acetaminophen), and other toxic compounds (mercuric chloride, carbon tetrachloride, uranium, cadmium, and chlorpryfos-ethyl) [100, 102].

Melatonin at the dose of 10 mg/kg/day along with colistin at the cumulative dose of 36.5 mg/kg could significantly mitigate the consequences of colistin-induced renal injury including increased urinary excretion of NAG and plasma Cr level as well as renal histological abnormalities. Melatonin also decreased the total body clearance and increased the half-life of colistin [103].

### N-Acetyl cysteine

N-Acetylcysteine (NAC), as a sulfhydryl-containing compound, is a precursor to the amino acid L-cysteine and consequently the antioxidant glutathione. NAC is found naturally in some vegetables and fruits, mostly in plants of the *Allium* species. It has mucolytic, antioxidant, anti-inflammatory, anticarcinogenic, and vasodilatory effects [104]. Due to the sulfhydryl group (–SH) within NAC, this molecule is able to scavenge free radicals, modulate cytokine synthesis through the inhibition of NF-κB, stabilize proteins and DNA structures, and chelate metals. NAC has been widely used in cystic fibrosis, acetaminophen overdose, and chronic obstructive lung disease. Previous studies have demonstrated the beneficial impact of NAC on non-alcoholic steatohepatitis, diabetic neuropathy, retinopathy, nephropathy, various types of cancer, infectious diseases (Acquired Immunodeficiency Syndrome (AIDS), tuberculosis, influenza, and diseases caused by respiratory syncytial virus, Helicobacter pylori, and SARS-CoV-2), stroke, Parkinson’s disease, dementia, recurrent unexplained pregnancy loss, male infertility, polycystic ovary syndrome, psychiatric disorders (schizophrenia, bipolar disorder, obsessive compulsive disorder, and addiction behavior), metal toxicity, age-related macular degeneration, cataract, and dry eye syndrome [105].

Recently, special attention has been drawn to NAC as a drug to attenuate contrast-induced nephropathy. Several studies have been conducted in this field, but the results are too conflicting to draw definite conclusions.[106]. The effect of NAC on renal function has also been evaluated in renal toxicity caused by cisplatin, ifosfamide, and renal ischemia–reperfusion injury [107–109].

Ozyilmaz et al*.* disclosed that the simultaneous administration of 150 mg/kg IP NAC with 300,000 IU/kg/day IP colistin for six consecutive days did not exert any significant effects on biochemical parameters including plasma BUN and Cr levels, creatinine clearance (ClCr), urine Cr and protein levels, TNF-alpha plasma level, and MDA tissue level. In contrast, reduction of elevated SOD levels and reversal of the increase in the tubular immunohistochemical expression of i-NOS and neurotrophin-3 were observed in NAC-treated rats [110].

In another study conducted by Ceylan et al*.* in 2018, NAC-induced protective and repairing effects against colistin nephrotoxicity were evaluated. The researchers found that the animals treated with 300,000 IU/kg/day CMS for 10 days showed marked elevations in Cr and urine NAG levels, while no significant changes were detected in serum Cr in the NAC + colistin group. Moreover, urine NAG levels were significantly higher in the CMS group compared to the other group on the 10th day of treatment. More interestingly, NAC could reverse colistin-induced negative effects, as determined by increased apoptosis index and renal histological damage score as well as by decreased renal expression levels of eNOS, SOD2, and matrix metalloproteinase (MMP). However, no significant differences were observed between the study groups regarding the renal total antioxidant and oxidant capacity. Thus, the researchers proposed that NAC exerted its beneficial effects through the activation of SOD2 and eNOS expression levels as well as MMP3 [111].

### Silymarin

Silymarin is extracted from the fruit and seeds of the *Silybum marianum* (milk thistle) and is a complex of other components such as a family of flavonolignans (silybin, isosilybin, silychristin, isosilychristin, and silydianin) and a flavonoid (taxifolin). Silybin is considered the major biologically active compound of the extract [112]. Silymarin has exhibited a number of pharmacological activities including antioxidant, immunomodulatory, antifibrotic, antiproliferative, anticancer, and antiviral properties. The cytoprotection activities of silymarin have been attributed to its antioxidant and radical scavenging effects. Silymarin has been used for centuries as a liver tonic and for the treatment of various liver diseases. Moreover, studies have demonstrated its protective effects in the fields of nephrotoxicity, hepatotoxicity, viral hepatitis, cancer, in vitro fertilization, neurotoxicity, depression, environmental toxins exposure, and lung, prostate, pancreas, and other diseases [113]. Silymarin has also shown protective roles in nephropathic processes including hemodialysis, end-stage diabetic nephropathy, contrast-induced nephropathy, renal injury caused by chemicals such as arsenic and ferric nitrilotriacetate, and drug-induced nephrotoxicity such as paracetamol, aminoglycosides, vancomycin, polymyxin, isoniazid, doxorubicin, cisplatin, cyclophosphamide, methotrexate, and cyclosporin [64, 114–116].

Hassan et al*.* evaluated the effect of silybin on the damages produced by polymyxin E on the rat kidney using histological, ultrastructural, and morphometric analyses. According to their findings, treatment with 50 mg/kg IV silybin in its active form (silymarin) twice a day for seven days two hours before each of the two daily doses of polymyxin E (at a cumulative dose of 36.5 mg/kg) alleviated the degenerative changes on rat kidney induced by polymyxin E. Interestingly, they observed a minimal residual pathology in animals treated with polymyxin + silybin, which suggested that the concomitant administration of silybin might not completely reverse the rapid process, by which polymyxin E induced nephrotoxicity [117]. Similarly, administration of 50 mg/kg IV silybin in the form of silymarin twice daily before IV polymyxin E (at the collective dose of 36.5 mg/kg) ameliorated the biochemical changes induced by polymyxin E in rats including elevated urinary NAG and serum levels of urea, Cr, UA, sodium, and potassium. However, the differences between the polymyxin and polymyxin + silybin groups were statistically significant only for NAG [118].

Recently, Dumludag et al*.* indicated that the co-administration of 750,000 IU/kg/day colistin and 100 mg/kg/day silymarin for 7 days led to a significant increase in GPx and SOD levels. In contrast, the study groups were not different in terms of serum MDA, Cys C, and Cr levels. Moreover, the results of histological examination demonstrated that silymarin led to some improvements in tubular necrosis. However, no significant changes were observed in the activity scores of tubular injury, medullar congestion, and interstitial inflammation and the apoptotic index [119]. The authors proposed that more pronounced nephroprotective effects were expected when silymarin was used at higher doses or in longer treatment courses.

### Taurine

Taurine is an amino acid found abundantly in most cells of the human body. Taurine has been confirmed to be a promising therapeutic agent against various disorders and pathological conditions due to its cytoprotective properties [120]. Various studies have been performed on the antioxidant activity of taurine in lung, liver, heart, and kidney. Studies have also emphasized the preventive and therapeutic effects of taurine therapy on the central nervous system (stroke, neurodegenerative diseases such as AD, Huntington, and Parkinson’s disease, epilepsy, and retinal degeneration), cardiovascular system (congestive heart failure, hypertension, atherosclerosis, ischemia–reperfusion injury, myocardial arrhythmias, metabolic diseases including mitochondrial diseases, diabetes, and arthritis), and muscles (sarcopenia, Duchenne muscular dystrophy, and myotonic dystrophy)[121]. Moreover, several studies have reported the beneficial effects of taurine on the kidney tissue in diabetic nephropathy, Fanconi anemia, cystinosis, chronic kidney disease, and acute kidney injury caused by lead, arsenic, acetaminophen, gentamycin, and radiation [122].

In a study carried out on colistin-treated mice (15 mg/kg/day IV for seven consecutive days), the effect of taurine was assessed on various plasma biomarkers of nephrotoxicity, kidney tissue markers of oxidative stress, and kidney mitochondrial indices. The results indicated that taurine administration (500 and 1000 mg/kg/day) intraperitoneally in combination with colistin for 7 days led to a significant decrease in colistin-induced elevation in the plasma levels of Cr and BUN. Taurine could also alleviate colistin-induced oxidative stress and mitochondrial dysfunction in the kidney tissue. Furthermore, taurine treatment reversed colistin-induced negative effects such as increased kidney ROS, lipid peroxidation (LPO), tissue oxidized glutathione (GSSG) level, mitochondrial LPO, permeabilization, and GSSG content, decreased renal tissue antioxidant capacity, and reduced glutathione stores, mitochondrial dehydrogenase activity, membrane potential, GSH, and ATP. Kidney tissue histopathological alterations were not evident in colistin-taurine groups. The regulation of mitochondrial function and decreased oxidative stress seem to be a fundamental mechanism of the positive effects of taurine on colistin nephrotoxicity [123].

### Vitamin C

Vitamin C is a water-soluble essential vitamin, which exists in the body primarily in its reduced form; i.e., ascorbic acid. Vitamin C is potentially involved in the biosynthesis of collagen and is a co-factor in the biosynthesis of catechol amines, L-carnitine, cholesterol, amino acids, and some peptide hormones. It is a chain-breaking antioxidant and free radical scavenger due to its ability to donate electrons and be readily converted back to its reduced form [124]. Vitamin C has shown beneficial effects on the prevention and treatment of several health conditions including scurvy, common cold, male and female infertility, atherosclerosis, cardiovascular diseases, cancers, diabetes, heavy metal toxicity (lead, arsenic, and cadmium), neurological and neurodegenerative disorders (schizophrenia, Parkinson’s disease, and AD), asthma, respiratory conditions, and ocular diseases (cataract and diabetic retinopathy). Moreover, this micronutrient can accelerate wound and burn healing, enhance immune system, and improve outcomes in critically ill patients [125]. Studies have also demonstrated the nephroprotective effects of vitamin C against nephrotoxicity caused by gentamicin, vancomycin, ampicillin, cisplatin, and contrast agents [126–128].

Yousef et al*.* conducted a dual in vivo and in vitro study to determine the protective effects of ascorbic acid against colistin-induced nephrotoxicity and apoptosis. In that study, ascorbic acid was administered at a dose of 50 or 200 mg/kg twice daily 20 min before each colistin dose (cumulative dose of 36.5 mg/kg) for 7 days. The results showed that urinary NAG excretion was significantly lower in the colistin/ascorbic acid 200 mg/kg group than in the colistin group. Additionally, no significant increase was observed in the plasma level of Cr in the colistin/ascorbic acid 200 mg/kg group compared to the colistin group. However, no significant differences were observed between the two groups in terms of SOD activity. Cell culture studies demonstrated that ascorbic acid had a dose-dependent inhibitory effect on colistin-induced apoptosis. Besides, histological examinations indicated the nephroprotective effect of ascorbic acid, at both low and high doses. More interestingly, ascorbic acid was able to decrease the total body clearance of colistin [128].

In an open-label, non-placebo, randomized controlled trial, Sirijatuphat et al*.* studied the potential nephroprotective effect of IV ascorbic acid against colistin-associated nephrotoxicity. In that study, 15 patients were given colistin alone (CMS at a loading dose of 300 mg of colistin base activity (CBA) followed by renally adjusted maintenance doses every 12 h), while 13 patients received a combination of colistin and IV ascorbic acid (2 g every 12 h, 20 min before colistin). The findings revealed no significant differences in the incidence of nephrotoxicity and the urinary excretion rates of urinary neutrophil gelatinase-associated lipocalin and NAG at various time points during colistin treatment, plasma colistin concentrations, patients’ clinical and microbiological outcomes, and mortality [129].

### Vitamin E

Vitamin E is a family of eight fat soluble compounds including a-, b-, g-, and d-tocopherol and a-, b-, g-, and d-tocotrienol, with α-tocopherol being the most widely known analog and being used most preferentially by the body. Vitamin E, primarily located in the cell and organelle membranes, can be found ubiquitously in various foods and different natural sources. It is considered a potent antioxidant, which can effectively scavenge free radicals and prevent LPO [130]. Vitamin E has been proven to exert excellent antioxidant, anti-inflammatory, anti-diabetic, anti-atherogenic, bone and joint protective, neuroprotective, skin protective, anti-obesity, immune-enhancing, and platelet aggregation inhibitory effects in different in vivo and in vitro studies. Potential therapeutic applications have also been suggested for vitamin E including Parkinson’s disease, AD, obesity, diabetes, atherosclerosis, osteoporosis, AIDS, rheumatoid arthritis, osteoarthritis, radioprotection, and different types of cancer [131]. Moreover, vitamin E has demonstrated promising properties in ischemia/reperfusion, cadmium, phenol, paraquat, contrast agents, aminoglycosides, cisplatin, doxorubicin, vancomycin-induced nephrotoxicity, and diabetic nephropathy [132].

The effect of vitamin E on colistin-induced nephrotoxicity was assessed by Ghlissi’s et al*.* Based on the results, treatment of rats with CMS at 300,000 IU/kg/day IM for 7 days led to a slight focal tubular dilatation, whereas the severity of renal damage was more prominent in the group receiving 450,000 IU/kg/day of CMS, causing acute tubular necrosis. The results also revealed a significant increase in the plasma levels of Cr, urine GGT, and MDA and a decline in the antioxidants’ activities including SOD, CAT, GPx, and GSH in the renal tissue following colistin treatment. Co-treatment with vitamin E at a daily dose of 100 mg/kg/day could partially prevent renal damage as well as changes in antioxidant parameters and LPO markers. These effects might be due to the scavenging activity of vitamin E against free radicals and the protection of cellular biomembrane from oxidative attack [43].

Ghlissi et al*.* conducted another study to determine the spontaneous renal recovery after stopping the low and high doses of colistin (300,000 IU/kg/day and 450,000 IU/kg/day of CMS for 7 days, respectively) as well as the curative effect of oral vitamin E (100 mg/kg/day for 2 weeks after colistin discontinuation) on antioxidant status and colistin-induced renal damage in colistin-treated rats. They found that vitamin E treatment improved tubular regeneration and normalized the altered biomarkers (including increased NAG and MDA levels and decreased SOD and GSH activities) in the colistin-treated rats. On the contrary, stopping CMS alone did not cause significant renal recovery [133].

The authors of the previous studies conducted another research and assessed the synergistic effect of vitamins E and C on colistin-induced nephrotoxicity in rats. The rats were exposed to 450,000 IU/kg/day of CMS for 7 days, which induced acute tubular necrosis, increased the urine NAG and GGT levels as well as renal tissue MDA levels, and reduced the plasma levels of vitamins E and C and renal tissue activities of SOD, CAT, and GPx. The findings demonstrated that the co-administration of vitamins E and C (at 100 mg/kg each) could improve the histopathological damage and restore all the mentioned biochemical parameters. The authors concluded that the combination therapy of vitamins E and C had a better antioxidant effect against colistin-induced tubular damage than either of the two vitamins given alone, which was attributed to their different subcellular locations as well as to their synergistic action [134].

In a pilot animal study, the nephroprotective effect of alpha-tocopherol, as the most common and biologically active form of vitamin E, was tested in colistin-treated rabbits. In that study, colistin was administered in low (an IV loading dose of 80 mg/kg CMS followed by IM maintenance doses of 10 mg/kg colistin sulphate for 6 days) and high (an IV loading dose of 120 mg/kg CMS followed by IM maintenance doses of 30 mg/kg colistin sulphate for 6 days) doses [135]. Oral α-tocopherol was administered at a dose of 200 mg for 1 week before colistin treatment and half an hour prior to exposing the animals to colistin in the subsequent week. The results showed a significant decrease in the serum level of Cr, as well as the attenuation of the pathological lesions in the kidneys following α-tocopherol administration. Tocopherol also significantly reduced the serum level of urea in the low-dose group [136].

A randomized clinical trial is being conducted in Loghman-Hakim hospital, a referral tertiary teaching medical center in Iran, to investigate the effect of a daily dose of 400 mg alpha-tocopherol on colistin nephrotoxicity [137].

## Conclusion

This review aimed to present an overview of different compounds used in experimental and clinical studies for preventing or attenuating colistin nephrotoxicity. These compounds including ABGE, albumin fragments, ALA, astaxanthin, baicalein, chrysin, cilastatin, colchicine, curcumin, cyt c, dexmedetomidine, gelofusine, GSPE, hesperidin, luteolin, lycopene, melatonin, NAC, silymarin, taurine, vitamin C, and vitamin E exhibited beneficial effects in most of the published works. These effects were attributed to their antioxidant, anti-inflammatory, and anti-apoptotic activities. However, the clinical experience about these agents was limited to only one clinical trial, in which the efficacy of IV ascorbic acid was evaluated in patients and the other studies were all animal-based studies. Thus, further clinical studies are required to elucidate the potential usefulness of these agents against nephropathy induced by colistin. Considering the availability in clinical practice, minimal adverse effect profiles, and positive results in animal studies, curcumin, melatonin, NAC, silymarin, and vitamin E can be suggested to be used in clinical trials.

There may be some possible limitations in this study. First, only English-language articles with full text were included in this searching strategy. Second, the majority of studies included in this review were animal studies, and the clinical use of nephroprotective agents was not evaluated. Another limitation is that the quality, number of sample sizes and studied parameters were various in the reviewed studies. Therefore, it is difficult to compare the treatment effects among these interventions.

## Data Availability

Not applicable.

## Abbreviations

AKI: Acute kidney injury
BUN: Blood urea nitrogen
CAT: Catalase
CBA: Colistin base activity
CMS: Colistimethate sodium
eNOS: Endothelial nitric oxide synthase
FRAP: Ferric reducing antioxidant power
GGT: Gamma-glutamyl transferase
GSH: Reduced glutathione
GSSG: Oxidized glutathione
HO-1: Heme oxygenase-1
iNOS: Inducible NO synthase
GABA: Gamma amino butyric acid
GPx: Glutathione peroxidase
IL: Interleukin
I.M.: Intramuscularly
I.P.: Intraperitoneally
K: Potassium
KIM-1: Kidney injury molecule-1
LPO: Lipid peroxidation
Na: Sodium
NAG: N-Acetyl-b-D-glucosaminidase
NGAL: Neutrophil gelatinase-associated lipocalin
NO: Nitric oxide
NF-κβ: Nuclear factor-κβ
Nrf2: Nuclear factor-erythroid 2-related factor 2
NT-3: Neurotrophin-3
OSI: Oxidative stress index
PCr: Plasma creatinine
ROS: Reactive oxygen species
S.C.: Subcutaneous
SCr: Serum creatinine
SOD: Superoxide dismutase
TAS: Total anti-oxidative stress
TNF-α: Tumor necrosis factor-α
TOS: Total oxidative stress
UA: Uric acid
UCr: Urine creatinine
8-OHdG: 8-Hydroxydeoxyguanosine
AIDS: Acquired immunodeficiency syndrome
ALA: Alpha-lipoic acid
AD: Alzheimer’s disease
ABGE: Aged black garlic extract
Bcl-2: B-cell lymphoma 2
COPD: Chronic obstructive pulmonary diseases
CBA: Colistin base activity
ClCr: Creatinine clearance
COX-2: Cyclooxygenase-2
Cys C: Cystatin C
cyt c: Cytochrome C
DHP-I: Dehydropeptidase-I
DNA: Deoxyribonucleic acid
iNOS: Inducible nitric oxide synthase
IL: Interleukin
LPS: Lipopolysaccharide
MDA: Malondialdehyde
MMP: Matrix metalloproteinase
mRNA: Messenger ribonucleic acid
MS: Multiple sclerosis
NAC: N-Acetylcysteine
NAG: N-Acetyl-β-D glucosaminidase
Nox4: NADPH oxidase 4
NO: Nitric oxide
NF-κB: Nuclear factor kappa-light-chain-enhancer of activated B cells
Akt: Protein kinase B
PTECs: Proximal tubule epithelial cells
TUNEL: Terminal deoxynucleotidyl transferase dUTP nick end labeling
TGF-β1: Transforming growth factor-beta 1
TNF-α: Tumor necrosis factor-alpha
TAS: Total anti-oxidative stress
–SH: Sulfhydryl group
